# Blood Glucose Levels and Performance in a Sports Camp for Adolescents with Type 1 Diabetes Mellitus: A Field Study

**DOI:** 10.1155/2010/216167

**Published:** 2010-08-02

**Authors:** Dylan Kelly, Jill K. Hamilton, Michael C. Riddell

**Affiliations:** ^1^School of Kinesiology and Health Science, Muscle Health Research Centre, Physical Activity and Diabetes Unit, York University, 4700 Keele Street, Toronto, ON, Canada M3J 1P3; ^2^Division of Endocrinology, Hospital for Sick Children, University of Toronto, Toronto, ON M551A1, Canada ON M551A1

## Abstract

*Background*. Acute hypo- and hyperglycemia causes cognitive and psychomotor impairment in individuals with type 1 diabetes mellitus (T1DM) that may affect sports performance. *Objective*. To quantify the effect of concurrent and antecedent blood glucose concentrations on sports skills and cognitive performance in youth with T1DM attending a sports camp. *Design/Methods*. 28 youth (ages 6–17 years) attending a sports camp carried out multiple skill-based tests (tennis, basketball, or soccer skills) with glucose monitoring over 4 days. Glucose levels at the time of testing were categorized as (a) hypoglycemic (<3.6 mM); (b) within an acceptable glycemic range (3.6–13.9 mM); or (c) hyperglycemic (>13.9 mM). *Results*. Overall, sports performance skill was *∼*20% lower when glucose concentrations were hypoglycemic compared to either acceptable or hyperglycemic at the time of skill testing (*P* < .05). During Stroop testing, “reading” and “color recognition” also degraded during hypoglycemia, while “interference” scores improved (*P* < .05). Nocturnal hypoglycemia was present in 66% of subjects, lasting an average of 84 minutes, but this did not affect sports skill performance the following day. *Conclusions*. Mild hypoglycemia markedly reduces sports skill performance and cognition in young athletes with T1DM.

## 1. Introduction

Youth with type 1 diabetes mellitus (T1DM) are regularly subjected to periods of both low (hypoglycemia) and high (hyperglycemia) blood glucose levels, as exogenous insulin therapy does not perfectly mimic endogenous insulin needs. The balance of glucose control is particularly challenging in young people with the disease since insulin requirements are influenced by a number of ever changing variables including nutritional intake, physical activity levels, and the circadian rhythms of other anti-insulin hormones. Moreover, any physical or emotional stress, sometimes associated with the stress of competition can increase glycemic excursions [1]. Indeed, the period of adolescence, perhaps because of both physiologic and behavioral factors, make the achievement of optimal glycemic control particularly challenging for youth with T1DM [2]. Regular physical activity (i.e., exercise), while overall has several beneficial effects for the child with T1DM, can make glycemic control very challenging [3]. Immediately following exercise, blood glucose concentrations may be low due to excessive insulin administration or may be high due to the effect of epinephrine release from the excitement and intensity of the exercise. A few hours later, the glucose levels typically fall because of elevated insulin sensitivity that helps replete glycogen stores. Unfortunately, the symptoms of low or high glucose levels are often masked by increased physical activity and sporting competition [4]. 

Early symptoms of hypoglycemia may include trembling (shakiness), an accelerated heart rate, sweating, and increased hunger. Unfortunately, these early warning symptoms are not always present in a person with diabetes who suffers from autonomic nerve dysregulation. Moreover, evidence suggests that even after a small number of repeated hypoglycemic events, or following prior bouts of exercise, the autonomic response to hypoglycemia is diminished in otherwise healthy persons with T1DM [5]. Symptoms of severe neuroglycopenia generally occur only when blood glucose levels are extremely low (<2.5 mM) or prolonged and can result in acute confusion, disorientation, and clumsiness [6], a situation that is very undesirable from a health and safety perspective. Symptoms of hyperglycemia, on the other hand, may cause fatigue, dehydration and blurred vision or may go completely unnoticed by the individual [7]. What is particularly challenging in recognizing hypo- and hyperglycemia in active adolescents with T1DM is that many of the symptoms are also associated with vigorous exercise (increased heart rate, sweating, shakiness, fatigue, dehydration, etc.), thus making increased glucose monitoring critical [3].

The degree to which acute or chronic hypoglycemia or hyperglycemia influence sports performance is unclear. Circumstantial evidence suggests that an increase in plasma glucose availability might improve the exercise capacity perhaps because more fuel is readily available for muscle contraction. Some performance-related studies document an ergogenic effect of carbohydrate rich sports beverages in nondiabetic athletes [8]. However, very few studies have been conducted in which exercise performance is examined during differing levels of blood glucose concentrations in those with T1DM [9, 10]. In one study, compared with hyperglycemia or euglycemia, exercise capacity was reduced and ratings of perceived exertion increased with hypoglycemia in group of youth with T1DM, although the exercise was always stopped by the research investigators rather than the subjects for safety reasons [10]. 

In this field study we measured the effects of concurrent and antecedent (nocturnal) blood glucose levels on sport performance in youth with T1DM participating in a one-week sports day camp. We hypothesized that, compared with euglycemia, both hypo- and hyperglycemic states would result in deterioration of sporting skill performance.

## 2. Methods

### 2.1. Participants

Children and adolescents aging 9–17 years were recruited from the 2009 York University Diabetes Sports Camp. To be eligible for the study, subjects had to be clinically diagnosed with T1DM and be on insulin therapy. Exclusion criteria were (1) another serious chronic illness impacting one's ability to perform the sporting activity; (2) a significant developmental delay with an inability to understand the testing protocol; (3) visual colour blindness or the use of medications (other than insulin) known to affect glycemic control. 

Twenty seven youth with T1DM (15 males, 12 females), 9–17 years of age, agreed to participate. This represents 90% of those approached for the study. The mean age of subjects was 11.4 ± 1.9 years, duration of diabetes ranging from 1–13 years. Twenty four of these individuals agreed to participate in the Stroop testing, and seventeen agreed to overnight continuous glucose monitoring (CGM). Since not all subjects were fitted with the CGM at once, due to the availability of units, the continuous glucose data shown below for days 2 and 4 of the camp are for *n* = 11 and *n* = 10 subjects, respectively. The study was approved by the York University Human Participants Research ethics board. 

### 2.2. Study Procedures

All data collection took place in a field study setting from Monday July 20th to Friday July 24th, 2009 at the sports camp. 

#### 2.2.1. Sports Skill Assessment

Sport skill testing took place repeatedly for all participants starting on the second day of camp (Tuesday) lasting until the last day of camp (Friday). All sport skills were demonstrated by certified coaching staff on the day before testing and participants were encouraged to practice in order to limit a learning effect. In all cases, participants were instructed as to how the sports skill scoring would be calculated prior to their first test. Participants were evaluated on tennis, basketball, or soccer abilities in a structured manner. Participants performed skill testing in one sport only (i.e., the sport that they chose to focus on for the entire week). Participants were brought to the assessment stations at random points during the camp day and completed an average of 6 ± 1 tests during the week. Tennis assessments took place on full sized courts and involved the instructor consecutively volleying 15 balls in a standardized manner and points were scored for volleys returned by the participant. Basketball testing took place inside a university gymnasium with subjects standing 13 feet from the backboard of the net. 

Participants were given one minute to complete up to 15 shots and scoring was based on the number of baskets made. The soccer assessment involved participants kicking 10 balls into a series of three different sized soccer nets. Balls were lined up 13′ away from and parallel to the goal line. Three points were awarded for balls scored into the smallest net, two points for balls into the medium sized net, and one point for balls into the large net. If a ball struck the frame of the medium or smallest size net, the ball was counted as if it had entered the net a size larger. Final scores were calculated by subtracting the time taken to kick all ten balls (in seconds) from points scored.

#### 2.2.2. Blood Glucose Measurements and Categories

For safety reasons, no subject was asked to perform a sport skill if they felt symptoms of hypoglycemia. In general, participants performed routine self-glucose monitoring before meals and sometimes (e.g., those on pump therapy) before snacks. In addition, if symptoms of hypoglycemia were noted by the campers at any time during the camp, blood glucose was immediately tested and treated as required (i.e., 15 grams of fast acting dextrose tablets- Dex4, Can-Am Care, Montreal, Qu). 

Blood glucose measurements were also taken and recorded immediately following all sports skill assessments using a standard glucose meter (One Touch, LifeScan Inc, New Brunswick, NJ). Thus, both the subjects and the coaching staff were blinded to the glucose levels at the time of skill evaluation. For all skills, scores were categorized into hypoglycemia (<3.6 mmol/L), acceptable glycemic range, (3.6–13.8 mmol/L), or hyperglycemia (≥13.9 mmol/L). These categories were set a priori. The hypoglycemic range was based on a recent article [11] recommending that ≤3.5 mmol/L is a clinically relevant definition of hypoglycemia. Acceptable glycemic ranges for exercise participation, for those with T1DM, are typically recommended to be between 4.0 and 13.9 mmol/L, but participation is deemed safe if individuals are hyperglycemic but without ketoacidosis [12]. In order to compare data across all sports, participant's scores were converted to a percentage of their own personal best.

#### 2.2.3. Nocturnal Hypoglycemia

Overnight hypoglycemia was measured using the Medtronic iPro continuous glucose monitoring device, which measures interstitial glucose levels every 5 minutes [13]. This technology uses a thin enzyme-coated electrode catheter inserted as a “sensor” just under the skin of the abdomen or the upper arm. The reaction between interstitial fluid glucose and glucose oxidase located on the electrode produces hydrogen peroxide. This reaction converts the interstitial glucose into an electrical current proportional to the glucose concentration at the site of the catheter insertion. The iPro device is small, inconspicuous, and can be worn in virtually all sporting activities including swimming. This particular “professional” CGM devise does not show “real time” glucose values but converts interstitial fluid glucose concentrations to whole blood glucose concentrations once it is removed (usually after 72 hours of data collection) and the information is downloaded. As such, subjects and the investigators are blinded to the sensor data while wearing it. This technology has previously been shown to have the capacity to detect both nocturnal glucose levels, while the individual is sleeping, and glycemic excursions associated with exercise [13]. Product specialists inserted the devices from the Monday to the Wednesday of the camp and instructed the subjects on proper protocol for calibration with their own blood glucose monitor. Data recorded from the iPro were analyzed from 10 pm to 6 am for assessment of nocturnal hypoglycemia. Nocturnal biochemical hypoglycemia was defined as any interstitial glucose concentration ≤3.9 mmol/L on at least two consecutive five minute averages. The glucose iPro reading at 9:30 pm on nights when nocturnal biochemical hypoglycemia occurred (if it did occur) was compared to the mean at the same time on nonhypoglycemic nights using a student's *t*-test. Hyperglycemia (≥13.9 mmol/L) was also documented from 10 pm–6 am in an identical way. Subjects recorded at least 4 capillary blood glucose measurements, as measured by the glucose monitors, per day during the period between 0900 h and 1600 h for calibration purposes.

#### 2.2.4. Stroop Test Assessment

Age appropriate commercially available Stroop Colour and Word Tests (PAR, Inc, Lutz, Fla) were used to assess cognitive processing at different blood glucose concentrations [14]. The Stroop test is a commonly used clinical tool that measures selective attention, speed of functioning, and other psychological capacities that are affected by attentional fatigue. During the test, participants are asked to recite the name of the color that a word is printed (e.g., “blue,” “green,” or “red” ink) and not the word itself, which is a name of a color (e.g., the word “red" printed in blue ink instead of red ink). Naming the color of the word during this “interference” takes longer and is more prone to errors than when the color of the ink matches the name of the color [15]. Scores are determines for reading ability, color recognition and interference, with the latter an index of the capacity to maintain attention during the interference caused by the color changes in the typed words. 

Stroop testing took place from Monday to Friday (inclusive) and subjects completed an average of 5 ± 1.4 tests, each test lasting ~2–5 minutes. Willing participants were randomly brought to the “sidelines” for testing during the sports camp. Subjects carried out blood glucose measurements on a glucometer immediately following testing. Reading, color naming, and interference scores were converted to T-scores for their age according to the Golden Stroop manual and then grouped into the same blood glucose ranges as sports performance and analyzed using the same methods.

#### 2.2.5. Statistics

Values are reported as means ± standard deviations. A Type 3 orthogonal one-way ANOVA was used to compare overall sporting skill scores and Stroop score (both expressed as a percent of personal best) across the three blood glucose categories (hypo, euglycemia, hyperglycemia), followed by a fisher-LSD post-hoc test if a main effect of blood glucose concentration was found. A paired student's *t*-test was used to compare the average for the percentages of personal best scores on days following a bout of nocturnal hypoglycemia to the mean following a night without nocturnal hypoglycemia. Statistical analysis was carried out using Statistica 6.0 statistical software package (StatSoft, Tulsa, OK).

## 3. Results

### 3.1. Sports Skills and Blood Glucose Levels

**Figure 1:**
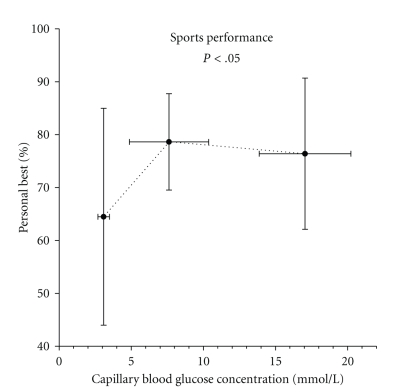
Sports skill performance score at each of the three categories of glycemia. Scores are shown as a percentage of personal best during the week. Scores during hypoglycemia were significantly lower than euglycemia or hyperglycemia. See methods section for a complete description. Mean ± SD. A significant main effect of glucose category on performance was found. Post hoc analysis reveled that performance in hypoglycemia was less than euglycemia and hyperglycemia at *P* < .05.

Sport skills were performed daily (Tuesday through Friday) by each subject at varying time points throughout the day, with an average of 6 ± 1 sports skill test done per subject over the course of the week, thus giving a total of ~160 sports skill tests. Because of the field study design, all subjects were tested while in acceptable glycemic range (*n* = 27), while only a portion of the subjects were tested while hypoglycemic (*n* = 7) or hyperglycemic (*n* = 10). The mean capillary blood glucose concentration during hypoglycemic testing was 3.1 ± 0.4 mmol/L, during the acceptable glycemic range was 7.6 ± 2.7 mmol/L, and during hyperglycemia was 17 ± 3.2 mmol/L. All data was expressed as a percentage of their personal best score to help account for the widely varying skill level among the subjects. Mean sport skill performance was highest when blood glucose values were in the acceptable glycemic range (3.6–13.8 mmol/L), with subjects performing 79 ± 9% (mean ± SD) of their personal best (Figure 1). Compared with the acceptable performance range, sports skill performance was lower during hypoglycemia (64 ± 20%, *P* < .05 versus euglycemia) but similar during hyperglycemia (76 ± 14). Of the 7 subjects tested while hypoglycemic, 1 subject scored his personal best during hypoglycemia. Three of 10 subjects performed best while hyperglycemic, while the remaining 24 subjects performed their personal best while in the acceptable glycemic range.

### 3.2. Daytime Glucose Concentrations as Measured by CGM

**Figure 2:**
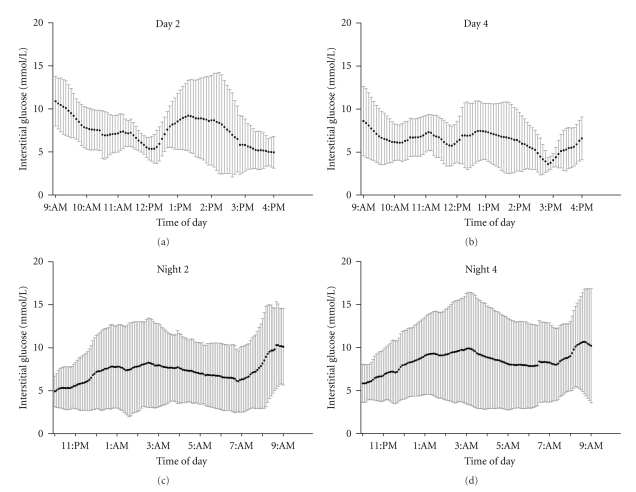
Interstitial glucose levels during day 2 ((a) *n* = 8) day 4 ((b) *n* = 10), evening 2 ((c) *n* = 8), and evening 4 ((d) *n* = 10) of the sports camp. Mean ± SD.

Interstitial glucose levels on day two and day four of the sports camp are shown in Figures 2(a) and 2(b), respectively. As expected, wide variation in glucose control was observed on both days. In general, mean glucose levels were largely within the acceptable range during the day but decreased during the morning activities. Glucose values increased post lunchtime and then decreased again in the afternoon. Four of eleven (36%) subjects had hypoglycemia (<3.6 mmol/L) at some point during the camp hours (9 AM–4 PM) on day 2 (9 AM–4 PM), while seven of ten (70%) subjects had hypoglycemia at some point during day 4. Duration of hypoglycemia ranged from 15 minutes to 2.5 hours.

In a separate analysis of iPro accuracy during the camp, 26 hypoglycemic glucometer readings were compared to the subject's corresponding iPro readings. The time difference between the two readings was always less than 2.5 minutes because the iPro produces a new reading every 5 minutes. The iPro overestimated glucose compared to the glucometer 13 times (50%), underestimated twice (8%), and was within 20% of the glucometer 11 times (42%).

### 3.3. Nocturnal Hypoglycemia as Measured by CGM

Figures 2(c) and 2(d) document nocturnal glucose levels during nights 2 and 4 of the camp. Table 1 shows the incidence of nocturnal hypoglycemia over the 4 nights of the camp. As shown in Table 1, nocturnal hypoglycemia often reoccurred in subjects. Of the 11 subjects with multiple overnight iPro data, 8 subjects (~73%) had multiple nights with hypoglycemic events, 1 subject (~9%) had a single night with hypoglycemia, while only 2 (18%) participants did not develop hypoglycemia. Forty one of the 45 nocturnal hypoglycemic events recorded had glucose levels <3.6 mmol/L, indicating that the incidence of moderate to severe hypoglycemia was common on the evening following sports camp participation. Twelve subjects with nocturnal hypoglycemic events completed sports skill testing on the day following the event compared with 9 subjects performing skill assessment on days without preceding nocturnal hypoglycemia. Performance did not differ between these conditions, including when blood glucose concentrations during assessments the following day were controlled for in the analysis.

### 3.4. Stroop Testing

**Figure 3:**
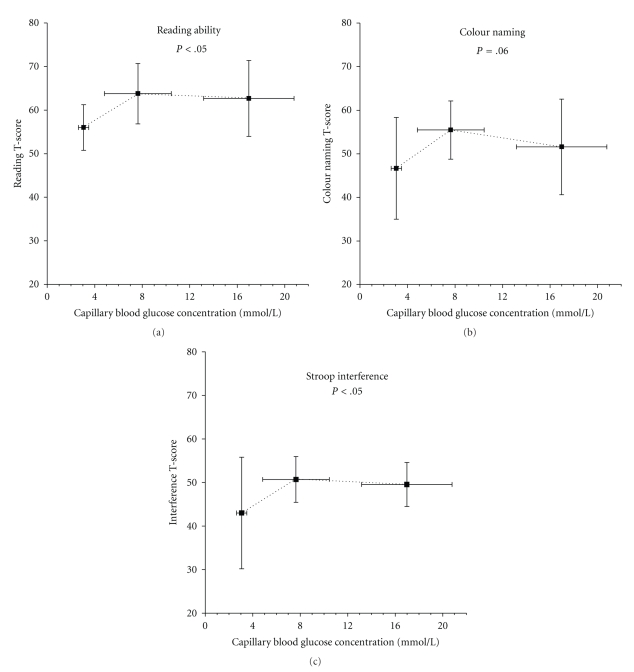
Stoop skill performance scores at each of the three categories of glycemia. Scores are shown as a percentage of personal best during the week. Scores during hypoglycemia were lower than euglycemia or hyperglycemia in reading ((a) *P* < .05), color recognition ((b) *P* < .05), and interference ((c) *P* = .06). See methods section for a complete description. Mean ± SD.

Stroop testing was done repeatedly on all subjects throughout the week. Because of the field study design, not all subjects were tested during hypo- (*n* = 7) and hyperglycemia (*n* = 9), although all 24 subjects were measured during the acceptable participation range. Reading ability was lower during hypoglycemia (56 ± 5% of personal best) than either euglycemia (64 ± 7%) or hyperglycemia (63 ± 9%, *P* < .05) (Figure 3(a)). Only 1 of 7 subjects read best during hypoglycemia, while 3 of 9 performed best during hyperglycemia. Similarly, color naming also tended (*P* = .06) to be lower during hypoglycemia (47 ± 12% of personal best) compared to either euglycemia (55 ± 7%) or hyperglycemia (52 ± 11%, Figure 3(b)). Two of 7 subjects had highest color recognition scores during hypoglycemia, while 6 of 9 had highest recognition during hyperglycemia. Interference score was also lower during hypoglycemia (43 ± 13%) but not during hyperglycemia (51 ± 8%), compared with euglycemia (50 ± 5%, *P* < .05, Figure 3(c)). Five of 7 subjects had their lowest interference score during hypoglycemia, while 5 of 8 had their lowest score during hyperglycemia.

## 4. Discussion

This field study, conducted in a unique sports skills camp for youth with type 1 diabetes, shows that the ability to carry out fundamental sports skills is markedly reduced by hypoglycemia compared with either euglycemia or hyperglycemia. This decrement in sports performance with hypoglycemia mirrors the decrement in cognitive performance in these adolescents, as assessed by the Stroop task protocol. In contrast to the finding that acute hypoglycemia influences performance, however, we found no evidence that a prior bout of nocturnal hypoglycemia influences sport skill performance the following day. This study is the first to examine sports performance associated with different levels of blood glucose levels in adolescents with type 1 diabetes. This is also the first study to measure nonendurance type sport performance in persons with type 1 diabetes.

Previously, we found that endurance cycling capacity is greater, and ratings of perceived exertion lower, when blood glucose levels are prevented from dropping to hypoglycemic ranges by providing exogenous carbohydrate in the form of a sports beverage [10]. However, our prior findings may have been contaminated by an order effect as exogenous glucose was always given during the second cycling test and the study investigators were not blinded to the blood glucose levels of the subjects, in part for ethical/safety reasons. In eight endurance-trained adults with T1DM, elevating blood glucose levels from 5.3 ± 0.6 mmol/L to 12.4 ± 2.1 mmol/L, via hyperinsulinemic glucose clamp technique, failed to change peak power output or other physiological endpoints such as lactate, heart rate, or respiratory exchange ratio [9]. Overall, these laboratory-based studies are limited; however, as performance was only measured during stationary cycling and not during sporting tasks that require a certain level of cognitive processing, reaction time, and motor skill performance. 

Hypoglycemia has long been thought to influence cognitive function in youth with diabetes. A recent field study of school-aged children with type 1 diabetes has shown that detrimental cognitive effects, as measured by math test performance, occurs when blood glucose levels are either hypo- or hyperglycemic [16]. Similar to their finding of an inverted U shape relationship between glycemia and cognitive performance, with the best performance in the euglycemic range, our study clearly demonstrated the negative effects of hypoglycemia on sports skill performance. Importantly, this finding of significantly impaired sports performance with hypoglycemia appeared universally across nearly all subjects, as only one subject performed better while hypoglycemia compared to euglycemia. Nonetheless, it is clear that the magnitude of the decrement of sport performance was highly individual with some subjects only showing minor reductions while others showing greater impairment. The reasons for the wide variation in sports skill performance during hypoglycemia are unclear but may be related to the level of blood glucose concentration, the rate at which glucose was dropping, the experience of the individual competing during hypoglycemia, or their capacity to maintain focus.

The detrimental effects of hypoglycemia on cognitive function are well documented in the literature [17]. We chose to make use of the child-specific Stroop test to asses the effect of glycemia on neuropsychological performance during the sports camp. The Stroop Test evaluates the ability to view complex visual stimuli and to respond to one stimulus dimension while suppressing the response to another dimension, an “executive” skill largely attributed to frontal lobe function [14, 15]. We found that reading and color recognition ability was more susceptible to impairment by hypoglycemia than interference score. In fact, we found that interference score was reduced during hypoglycemia compared with either euglycemia or hyperglycemia, indicating that hypoglycemia improved the capacity to maintain concentration during interference. An increased interference effect is found in disorders such as brain damage, dementias, and other neurodegenerative diseases, attention-deficit hyperactivity disorder, and a variety of mental disorders such as schizophrenia, addictions, and depression. As pointed out above, a lower interference score would usually be indicative of greater attention, however, given the neural insult of hypoglycemia this interpretation is likely not the case. A possible explanation for reduced interference suggested by the Stroop test is that there is poor dominance of the word naming system over the color naming system [14]. Although both color naming and word processing were impaired during hypoglycemia, the insult to the word processing system may be greater. This hypothesis requires further investigation, however.

Studies analyzing the cognitive impairment associated with hyperglycemia typically see a decline in ability at blood glucose levels >20 mmol/L [16]. Our failure to observe a similar deterioration in sports skill performance with hyperglycemia may have been due to the fact that the mean blood glucose concentration in our subjects were lower (16.9 ± 3.17 mmol/L) than what had been tested with cognitive function previously. Interestingly, a prior study in adults with type 1 diabetes found no change in endurance performance with mild hyperglycemia (~12 mmol/L) compared with euglycemia [9]. It is important to note that the hyperglycemia observed in our subjects was typically transient in nature, as evidenced by the CGM tracings, and perhaps a result of brief elevations in circulating catecholamines associated with the exercise itself. Indeed, it is likely that performance skill would decline if the level of hyperglycemia was higher or for a prolonged duration (days rather than minutes to hours), since individuals would be expected to be suffering from dehydration, ketosis, and reduced muscle and liver glycogen content [18]. However, this hypothesis requires further investigation.

Whether nocturnal glycemia influences either cognitive or sports performance the next day remains unclear. Previously, cognitive tests based on acute stimulus processing have failed to find a detrimental effect of preceding nocturnal hypoglycemia on cognition [19, 20]. Similarly, unlike concurrent hypoglycemia, we found that nocturnal hypoglycemia did not affect sports skill performance the following day. Even if sport skill performance is not influenced by antecedent hypoglycemia, *per se*, it is important to note that endurance exercise capacity may be impaired because of a higher risk for autonomic dysfunction and repeat hypoglycemia [21]. Future investigation is required, however, to compare endurance exercise performance using some measure of exercise capacity after a night of hypoglycemia versus euglycemia.

We found that the iPro device tended to overestimate blood glucose concentrations when subjects were deemed to be hypoglycemic, as measured by capillary finger stick/glucometer readings (i.e., 50% of the time in 26 paired valued). This overestimation in blood glucose levels with CGM was likely due to the lagging of sensor reported glucose changes behind blood glucose changes during exercise, as has been reported previously [22, 23]. It is worth noting that blood glucose meters also have approximately a 20% variance compared to plasma glucose as measured by a clinical devise, such as a YSI analyzer, and these paired values would be susceptible to variance in both the iPro and glucometer readings. Importantly, unlike the iPro devise used in this study, other “real-time” CGM devises have live glucose display and hypoglycemic alarm functions that can be set to alert the users when their sensor measures a certain glucose concentration. It is important to note that if the threshold for hypoglycemia in the real-time CGM units is set at 5.0 mmol/L, rather than 3.9, then, based on our study, ~85% of the hypoglycemic events would be caught by the sensor. 

This study has a number of important limitations that should be pointed out. First, the sports skill tests conducted were of relatively short duration (~1-2 minutes) and thus do not demonstrate how blood glucose levels affects mental and physical stamina (i.e., endurance capacity). Second, because of the field test design of the study, few subjects were testing in all three conditions (hypo, euglycemia, and hyperglycemia) and thus a repeated measured design could not be used for the analysis. Future studies may wish to clamp blood glucose levels at varying levels (hypoglycemia, euglycemia, and hyperglycemia) in a counterbalanced design so that a repeated measures design approach could be performed. 

In summary, we found that hypoglycemia, but not hyperglycemia, impairs sports skill performance and cognitive function in youth with type 1 diabetes. In contrast, prior exposure to hypoglycemia the night before competition does not appear to influence performance the following day. As such, vigilance in glucose control that limits hypoglycemia during sport should maximize competitive capacity in adolescents with type 1 diabetes. In addition, any obvious decrement in sport performance, such as poor passing, failed free throws, serves, and so forth; should be a warning sign to young athletes with diabetes to check for hypoglycemia by monitoring their blood glucose levels and treat hypoglycemia with additional carbohydrate intake.

## Figures and Tables

**Table 1:** Occurrences of nocturnal biochemical hypoglycemia during the evenings of the sports camp.

Night	Subjects (*n*)	Ratio of participants with nocturnal hypoglycemia	No. of nocturnal hypoglycemic events	No. of subjects with multiple nocturnal hypoglycemic events	Duration of nocturnal hypoglycemic events in minutes (mean ± SD)	Median duration of nocturnal hypoglycemic events in minutes
1	14	8/14	12	3	98 ± 116	50
2	8	5/8	11	4	97 ± 124	35
3	12	9/12	16	3	70 ± 92	30
4	8	5/8	6	1	89 ± 100	43
